# Supplementary material for: Reciprocal H3.3 gene editing identifies K27M and G34R mechanisms in pediatric glioma including NOTCH signaling
Source: Commun Biol. 2020 Jul 9;3:363. doi: 10.1038/s42003-020-1076-0 (PMC7347881; doi:10.1038/s42003-020-1076-0)
Supplement: Supplementary file 1 — Supplementary Information [file 42003_2020_1076_MOESM1_ESM.pdf]

Supplementary Information

Reciprocal H3.3 gene editing identifies K27M and G34R mechanisms in pediatric glioma including NOTCH signaling

Chen et al. 2020

Supplementary Figures

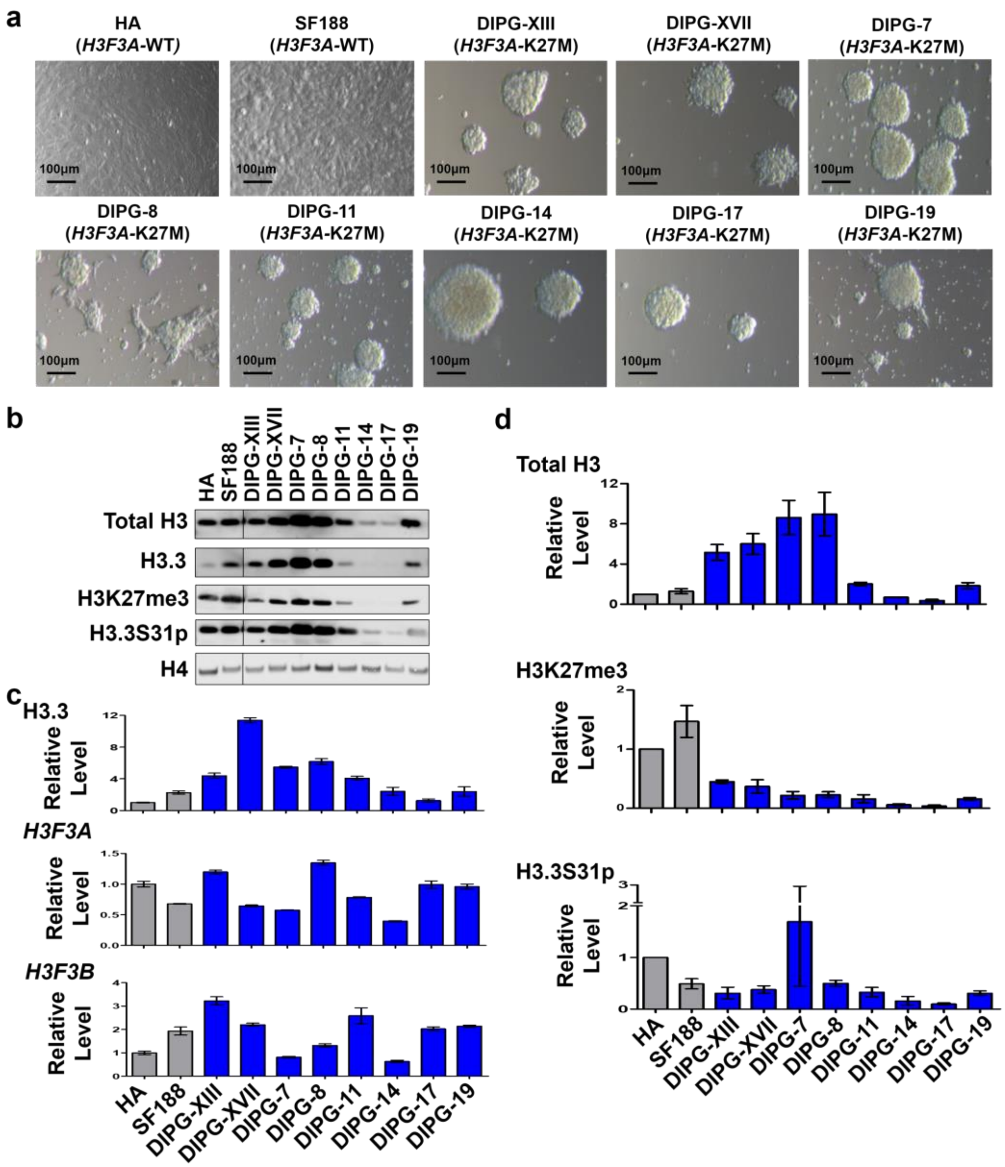

Supplementary Figure 1. H3F3A-mutant pediatric gliomas exhibit unique gliomatypic signatures. (**a**) Representative images of H3F3A-WT control lines (HA, SF188) and H3F3A-K27M pediatric DIPG cell lines described in this study. (**b**) Representative Western blot images for H3/H3.3 proteins as well as select H3 and H3.3 histone marks in pediatric glioma and control cell lines. In all blots for each antibody, the entire cell panel was run on the same gel/membrane, and boxes around lanes denote lanes that were empty or that contained ladder, which were omitted. (**c**) Quantification of H3.3 protein levels by Western blot as well as H3F3A and H3F3B RNA levels by qPCR. (**d**) Quantification of total H3, H3K27me3, and H3.3S31p levels by Western blot. H3K27me3 and H3.3S31p levels are normalized to total H3 and H3.3 levels, respectively. For Western blot quantifications, data are plotted as mean±sd fold expression relative to HA with n=2. RNA levels are plotted as mean±sd fold expression relative to HA with n=3. H3F3A-WT lines are plotted in gray and DIPGs with H3F3A-K27M mutation are in blue.

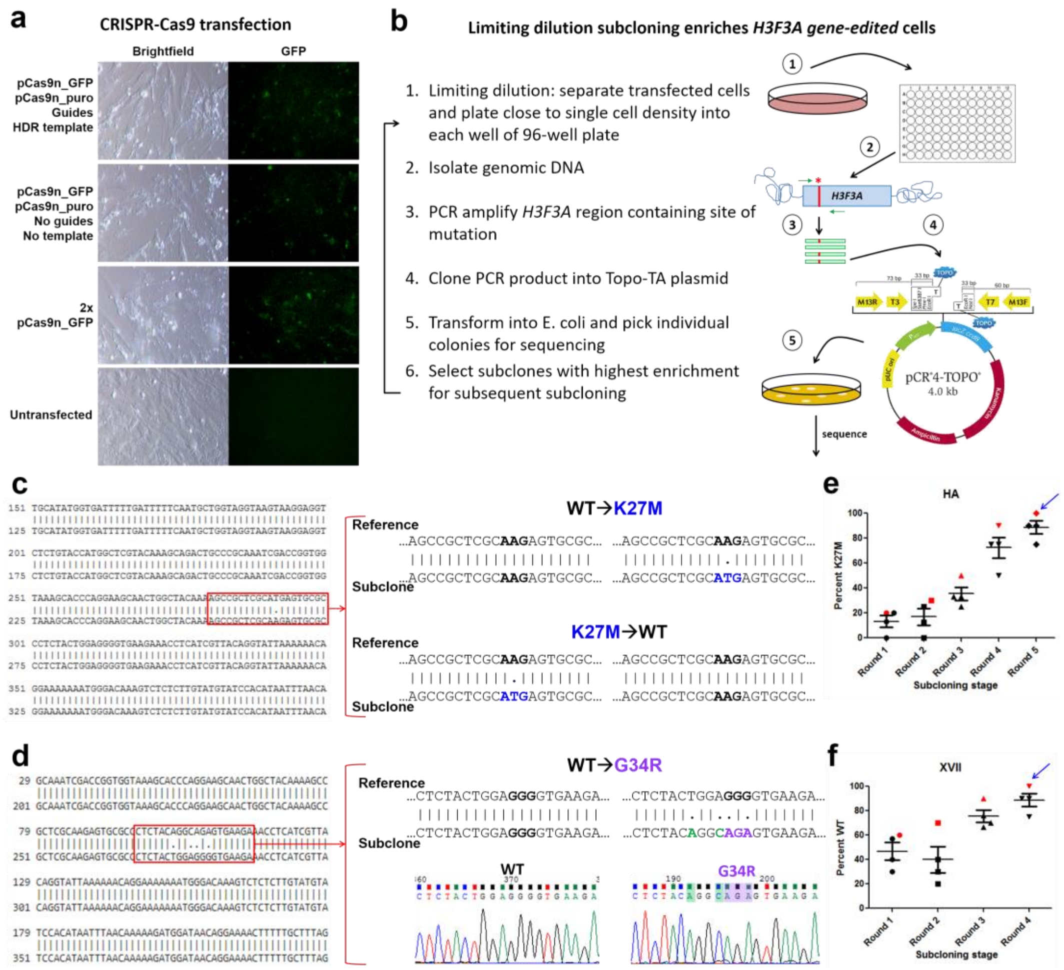

Supplementary Figure 2. CRISPR-Cas9 gene editing and subcloning to produce pairs of isogenic glioma lines distinguished only by their H3.3 mutation status. (**a**) Representative images depicting the transfection of HA cells with CRISPR-Cas9 gene editing components that include two Cas9-nickase plasmids containing guide sequences targeting *H3F3A* (pCas9n_GFP and pCas9n_puro) and homology directed repair (HDR) template containing the targeted K27M mutation. Control transfections contained combinations of no guides and no template as well as empty pCas9n_GPF with no guides. Untransfected cells are shown in comparison. G34R gene edits and conversions of mutant alleles to WT were conducted similarly. (**b**) Limiting dilution subcloning methodology to obtain clonal gene-edited cell lines. (**c, d**) Sequencing of *H3F3A* confirms K27M and G34R gene editing (blue and purple, respectively). Silent mutations (green) were introduced via our HDR template to prevent guide binding after successful editing. At least 10 sequences per subclone were obtained using the Topo-TA cloning and sequencing method; representative chromatograms are shown. (**e, f**) Example subcloning progression for HA and XVII lines in which iterative limiting dilution facilitated enrichment of desired mutations. Scatter dot plots with average and SEM is depicted for each round of subcloning in which four subclones were sequenced and percent mutation calculated from Topo-TA sequencing results. Each datapoint represents a different subclone well. The subclone with the highest percentage of the desired mutation (red datapoints) was expanded and used in subsequent rounds of subcloning until 100% of sequenced alleles contained the target gene edit (blue arrows).

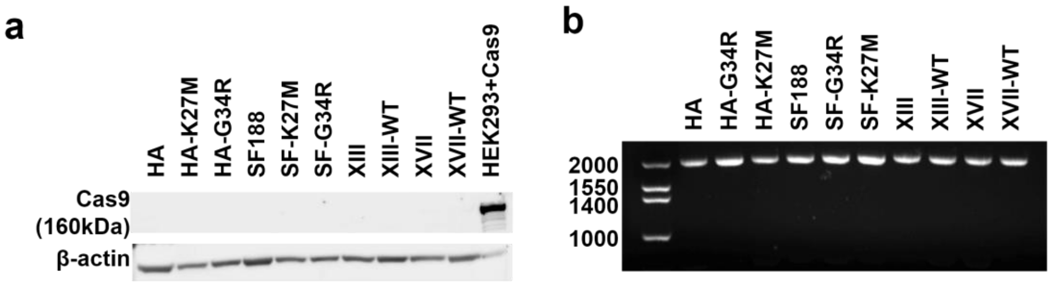

c

Predicted off-targets guide 1

chr	strand	position	sequence	n_mismatches	score	ontarget	gene
chr11	1	11878367	CTTCCTGTGTGCTTTACCACTGG	1	100	False	None
chr13	-1	72249229	CTTCCTGGGTGCTTTACCACCGG	0	100	False	None
chr2	-1	175584669	CTTCCTGGGTGCTTTACCACCGG	0	100	False	None
chr4	1	140619651	CTTCCTGGGTGCTTTACCACCGG	0	100	False	None
chr5	1	138991932	CTTCCTGGGTGCTTTACCACTGG	0	100	False	None
chr17	-1	26743961	CTCCCTGGGTGCTTTACCACAGG	1	98.6	False	None
chr9	1	26880490	CTTCCTGGATGCTTTACCACCAG	1	61.1	False	None
chr1	1	93214791	CTTCCTGGGTGTTTTACCACTGG	1	49.2	False	None
chr4	-1	113486177	CTTCCTGGGTGGTTTACCACCGG	1	49.2	False	None
chrX	-1	50648471	CTTCCTGGGTGCTATACCACCGG	1	14.9	False	None
chr18	1	34961809	CACCCTGAGTGCTTTACCACTGG	3	2.507898	False	None
chr5	-1	115106316	CTTTCTGGGTGCTTCACCACCGG	2	2.496078	False	None
chr1	1	53409547	CTTCCTGGATGCTTTACCAGTGG	2	2.373016	False	None
chr9	-1	1011118	CTTCCTAGGTGCTTTACCCCCAG	2	2.174338	False	None
chrX	1	28677529	CTTCCCGGGTGTTTTACCACCAG	2	1.991387	False	None
chr18	-1	74687744	CTGCCTGCCTGCTTTACCACAGG	3	1.532326	False	None
chr15	-1	94711609	CTTCCTGGGGGCTTTGCCACTGG	2	1.059799	False	None

Predicted off-targets guide 2

chr	strand	position	sequence	n_mismatches	score	ontarget	gene
chr1	-1	24275809	AAGTGCGCCCTCTACTGGAGGGG	1	100	False	None
chr13	1	72249278	GAGTGCGCCCTCTACTGGAGGGG	0	100	False	None
chr2	1	175584718	GAGTGCGCCCTCTACTGGAGGGG	0	100	False	None
**chr3**	**-1**	**109128829**	**GAGTGCGCCCTCTACTGGAGGGG**	**0**	**100**	**False**	**NM_001145553**
chr4	-1	140619602	GAGTGCGCCCTCTACTGGAGGGG	0	100	False	None
chrX	-1	28677480	GAGTGCGCCCTCTACTGGAGGGG	0	100	False	None
chrX	1	50648520	GAGTGCGCCCTCTACTGGAGGGG	0	100	False	None
chrX	1	122648402	GAGTGCGCCCTCTACTGGAGGGG	0	100	False	None
chr15	1	45507824	GACTGCGCCCTCTACTGGAGGGG	1	98.6	False	None
chr1	-1	93214742	GAGTGTGCCCTCTACTGGAGGAG	1	60.5	False	None
chr4	1	113486226	GAGTGTGCCCTCTACTGGAGGGG	1	60.5	False	None
chr3	-1	179367553	GAGTGCGCCCTCTACTGGACGGG	1	41.7	False	None
chr5	1	87898668	GAGTGCGCCCTCTACTGGACGGG	1	41.7	False	None
chr3	-1	25482920	GAGTGCGCCCTCTACTGGCGGGG	1	31.5	False	None
chr6	1	156983266	GAGTGCGCCCTCTACCGGAGGGG	1	17.2	False	None
chr11	-1	89232187	AAGTGCACCCTCTACTGGAGGGG	2	4.569366	False	None
chr6	-1	26322402	GAGTGCTCCCACTACTGGAGGGG	2	2.279188	False	None
chr15	-1	40243872	GACTGCACCTTCTACTGGAGGAG	3	1.616528	False	None
chr2	-1	209331261	CAGTGTGCCATCTACTGGAGGGG	3	1.52769	False	None
chr17	1	58393274	GAGTGCACCCTCTATTGGAGGGG	2	1.380094	False	None
chr1	-1	53409498	GAGTGTGCCCTCTACTGCAGGGG	2	1.198415	False	None

# of overlapping off-targets = 0

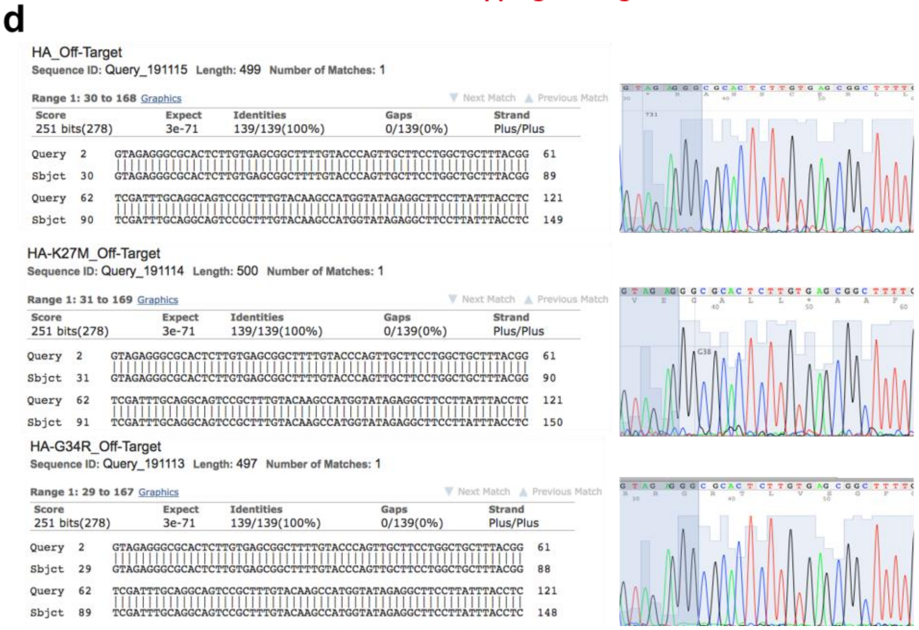

Supplementary Figure 3. Cas9 is transiently expressed after CRISPR transfection with no onsite Indels or detectable effect on predicted off-targets. (**a**) Western blot with anti-Cas9 indicates little to no persistent Cas9 expression in gene-edited cells subcloned via limiting dilution. A positive control sample of HEK293T stably transfected with Cas9 was included. (**b**) PCR amplicons of a 2 kb region of *H3F3A* gDNA spanning the targeted cut sites across our cell line panel shows that unintended insertions/deletion edits did not occur upstream or downstream of the cut sites. (**c**) Genes predicted by CRISPR guide design server (crispr.mit.edu) to be the relative top possible off-targets for each of the two guides separately (there were no predicted overlapping off-targets for both guides) used with Cas9-nickase for gene editing. NM_001145553.1, while initially listed as an exonic transcript was removed from the NCBI database. (From NCBI: "*RefSeq was permanently suppressed because currently there is support for the transcript but not for the protein.*") (**d**) Sanger sequencing of PCR amplified portion of potential off-target NM_001145553.1 gDNA suggests no indels or mutations induced. Sequencing was performed for all CRISPR-edited cell lines and example alignments and chromatograms (visualized using 4Peaks software) of the predicted potential off-target binding regions are shown.

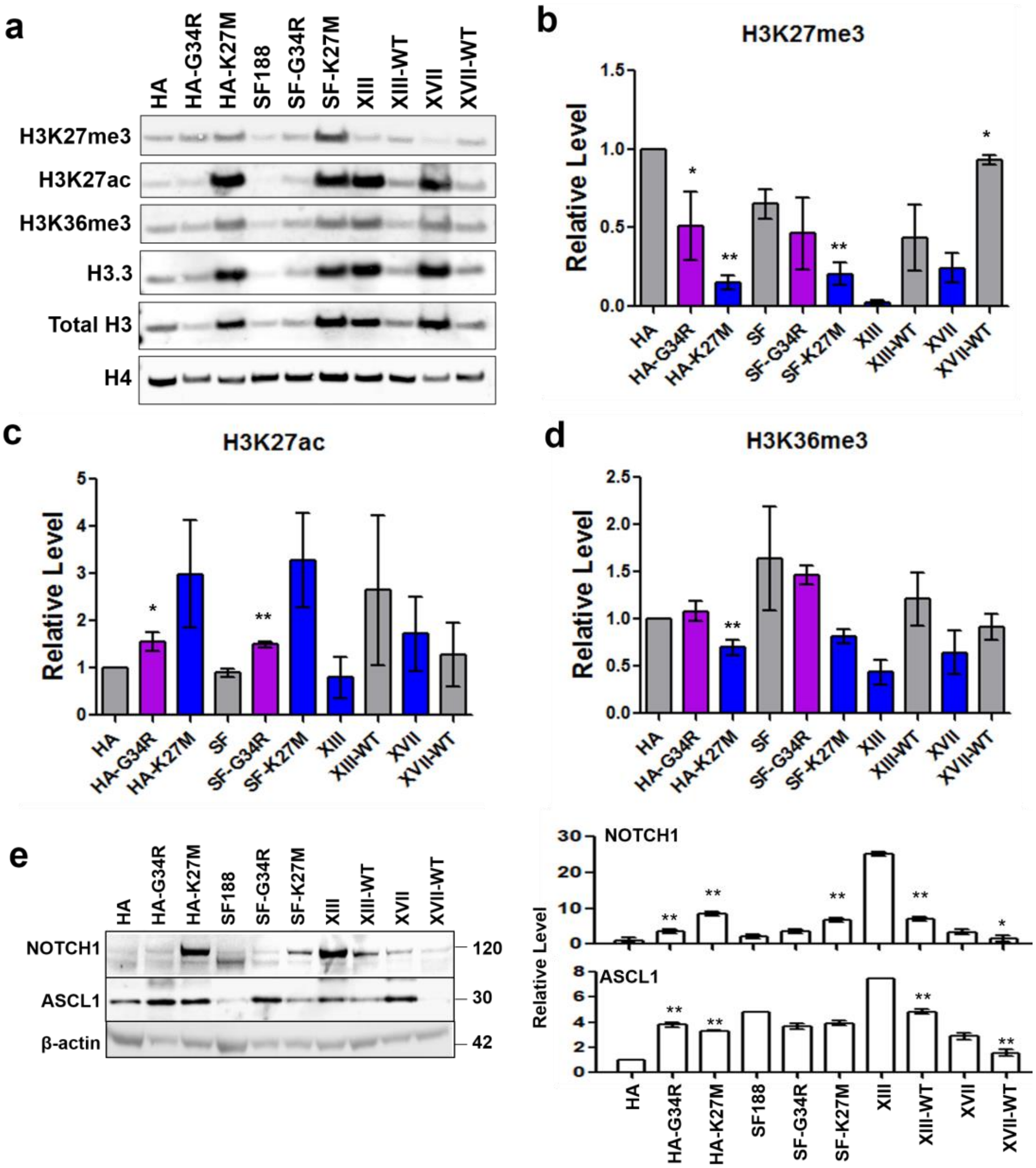

Supplementary Figure 4. Mutant H3.3 cell lines exhibit changes in histone H3 marks and NOTCH pathway protein levels. (**a**) Representative Western blot images for levels of H3.3, total H3, H4 and histone H3 marks for cell panel. Acid extracts enriched for histones were used. (**b-d**) Quantification of (**b**) H3K27me3, (**c**) H3K27ac, and (**d**) H3K36me3 levels for WT (gray), G34R (purple), and K27M (blue). Histone mark data are normalized to total H3 levels. (**e**) Representative Western blot images for NOTCH1 and ASCL1 in nuclear fractions of the various cell lines. Running sizes (kDa) on 4-12% gradient gel are listed. Quantification of NOTCH and ASCL1 levels are normalized to beta-actin and additionally to levels in HA cells. Data are plotted relative to HA as mean ± sd with n=5 for H3K27me3 and n=3 for H3K27ac and H3K36me3, n=2 (biological duplicate) for NOTCH1 and n=4 for ASCL1. *p<0.05, **p<0.01 for two-tailed student t-test compared to matched parental cells.

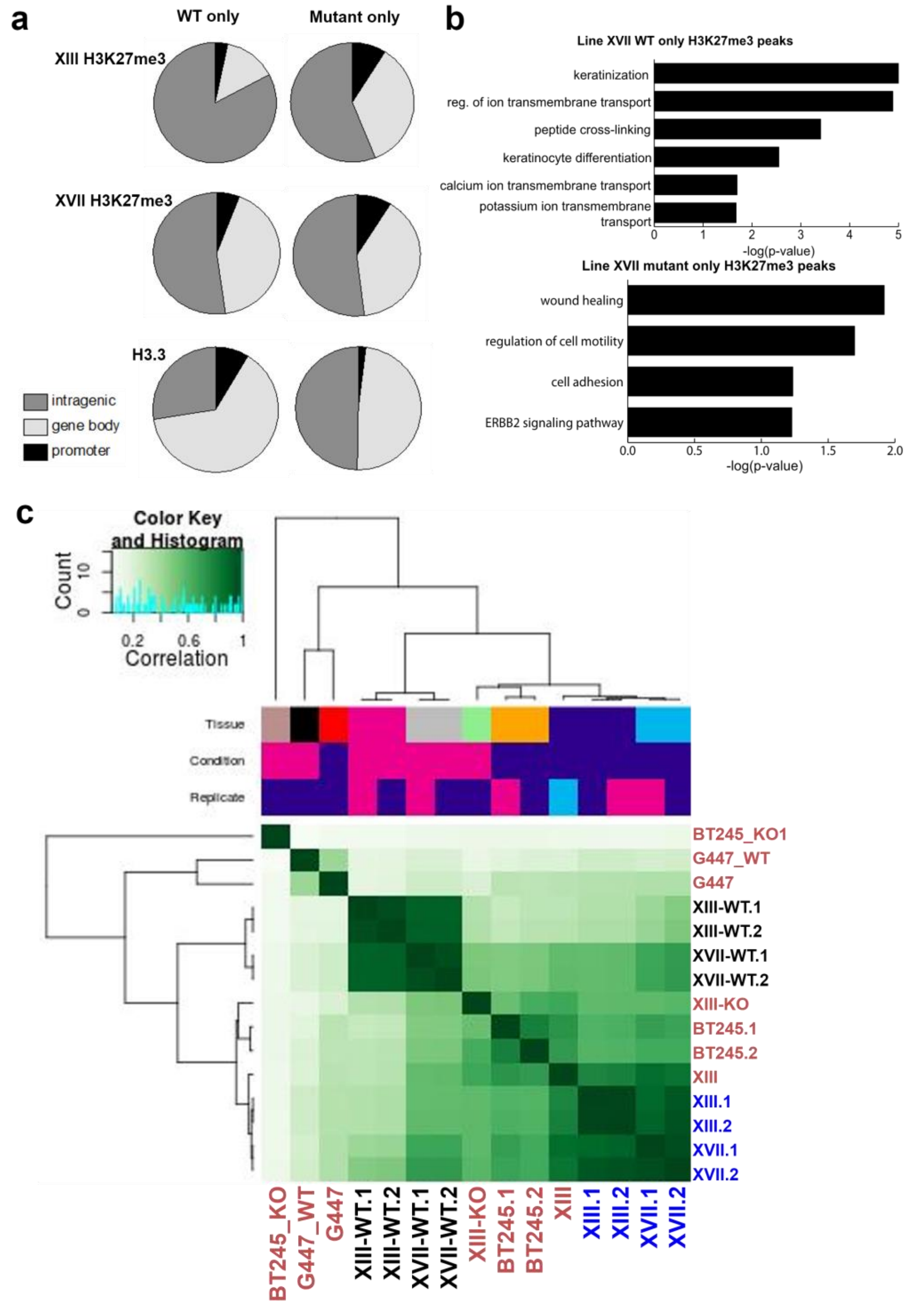

Supplementary Figure 5. Analysis of H3K27me3 and H3.3 ChIP-Seq data in XIII, XIII-WT, XVII, and XVII-WT cells. (**a**) Genomic distribution of unique H3.3 and H3K27me3 peaks in H3.3WT and K27M cell lines. (**b**) Gene ontology analysis of genes linked to H3K27me3 peaks with significant differential binding between XVII and XVII-WT as identified by the R package DiffBind. (**c**) Clustering of H3K27me3 ChIP of our cell samples (black, H3.3-WT; blue H3.3K27M) along with those from Harutyunyan et al. (orange)[1]. While our XIII and XVII parental and gene-edited lines were compared in duplicate, only BT245 from Harutyunyan et al. was available in duplicate, and the other cell lines are compared as single samples.

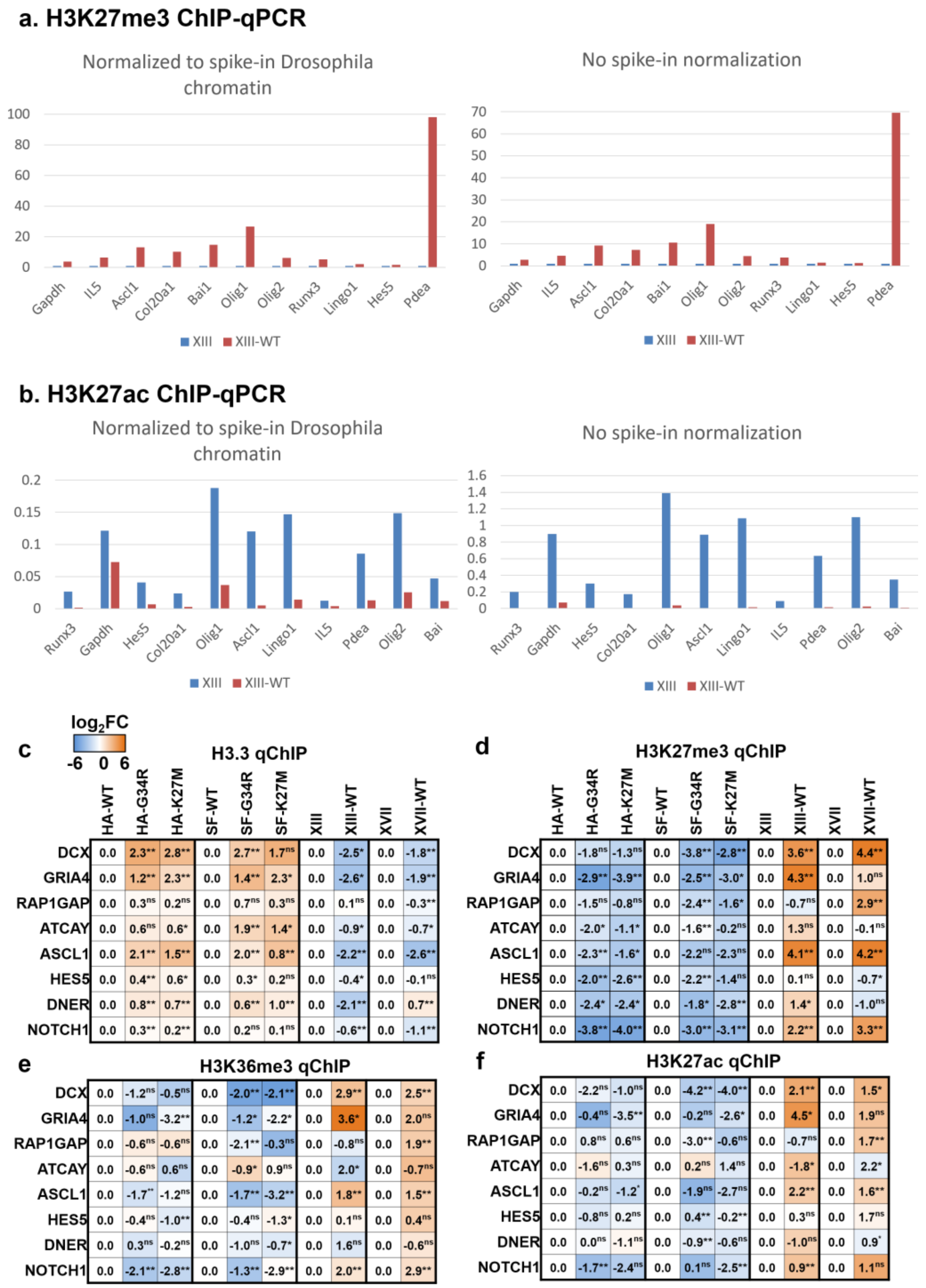

Supplementary Figure 6. H3.3 and histone mark levels at specific gene promoters track with H3.3 mutation status. (**a, b**) Comparison of H3K27me3 and H3K27ac levels at various selected gene regions by ChIP-qPCR either with or without spike-in Drosophila chromatin for normalization. (**c-f**) Heatmaps of qChIP data for (**c**) H3.3, (**d**) H3K27me3, (**e**) H3K36me3, and (**f**) H3K27ac in the promoters of select neurogenesis and NOTCH pathway genes tabulated as log_2FC relative to parental cell lines. H3K27me3, H3K36me3, H3K27ac values are normalized to total H3 levels. **p<0.05, **p<0.01, ns=not significant for two-tailed student t-test. Data shown are the means of n=3 (biological replicates).

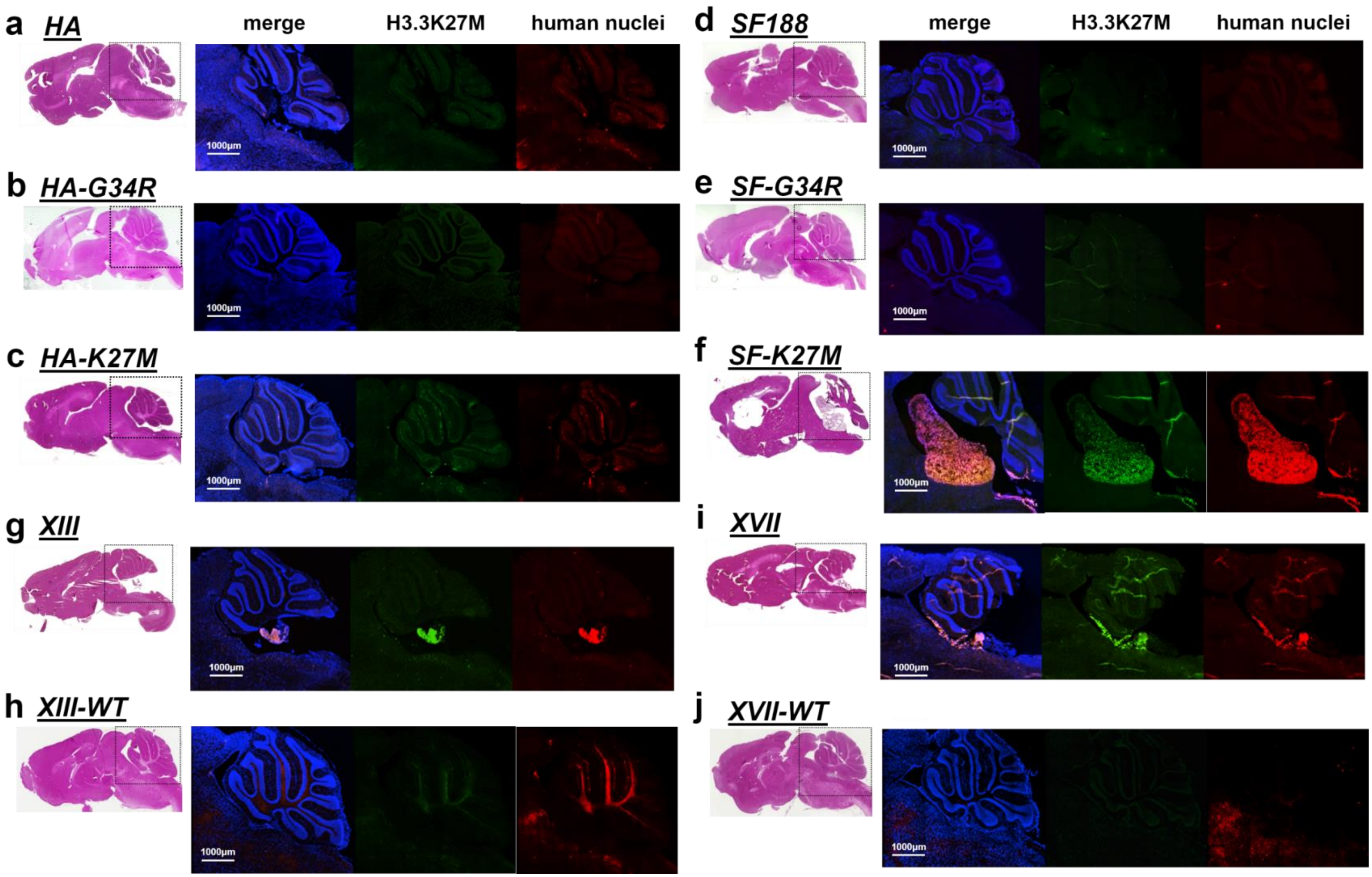

Supplementary Figure 7. Engraftment of tumor cells into mouse brain. (**a-j**) Representative hematoxylin and eosin and immunofluorescence (IF) stained images of mice brains injected with (**a**) HA, (**b**) HA-G34R, (**c**) HA-K27M (**d**) SF188, (**e**) SF-G34R, (**f**) SF-K27M, (**g**) XIII, (**h**) XIII-WT, (**i**) XVII, and (**j**) XVII-WT cells. IF staining with anti-human nuclei antibody (red) overlaid with anti-H3.3K27M staining (green) and DAPI staining (blue) confirms engraftment and growth of injected cells in the base of the cerebellum and in the pons. IF images are shown at 10X magnification and dotted boxes denote magnified regions. Xenograft injections for each cell line was performed with n=6 independent mice with equal gender ratio.

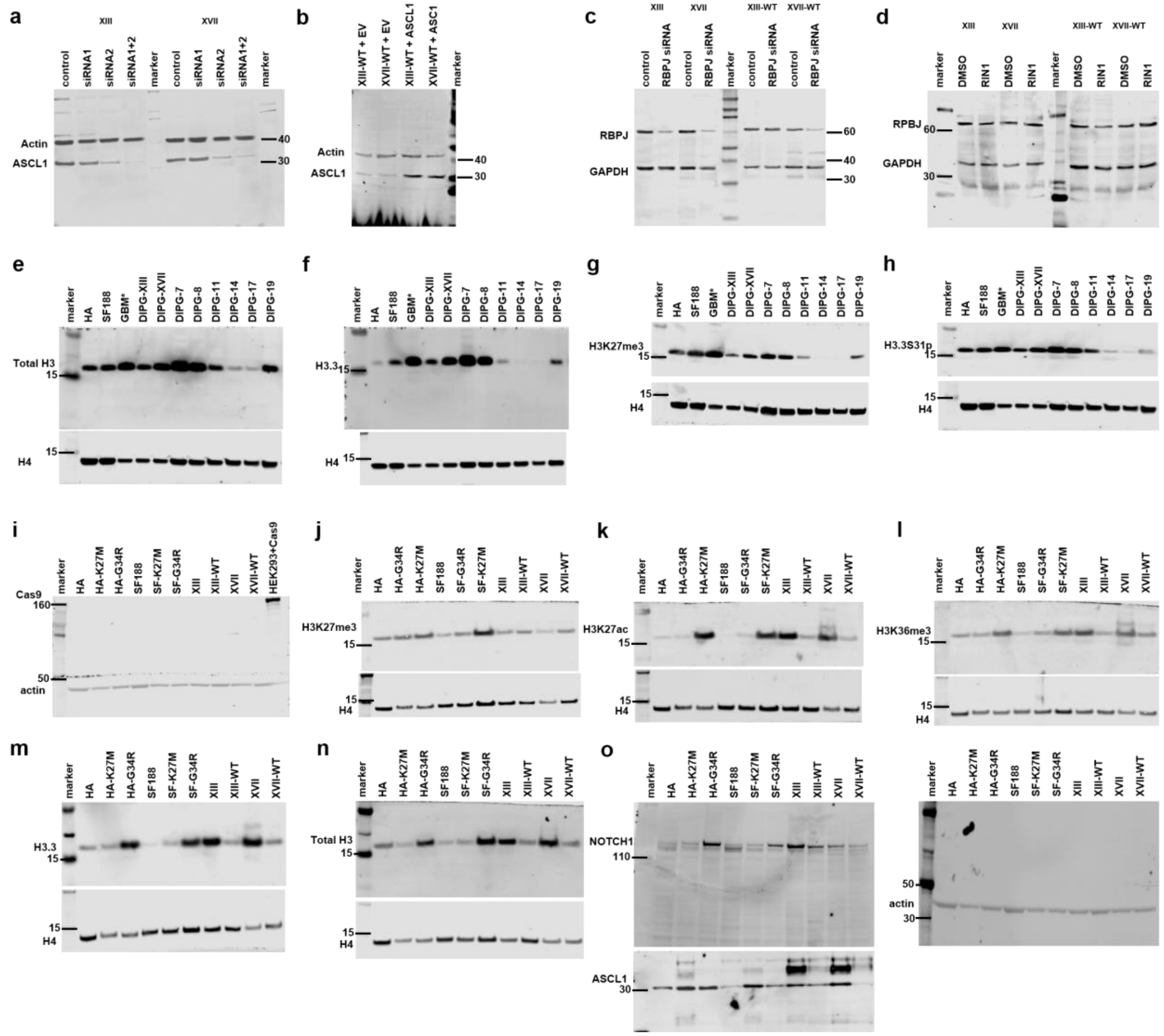

Supplementary Figure 8. Full uncropped Western blot images for all blots in main and supplementary figures. (**a-d**) Uncropped Western blots of the blots shown in Main Fig. 5c, e, g, and i, respectively. (**e-h**) Uncropped Western blots of blots shown in Supplementary Fig. 1b. Blots were cut after transfer and incubated with antibodies followed by imaging on Licor Odyssey. Reprobing of blots with different antibodies for different histones/histone marks where antibodies from different species were available was performed. *Note that the GBM cell line was blotted, but not discussed or included elsewhere in the study. (**i**) Western blot assessing whether there was persistent Cas9 expression in gene-edited cells after all the subcloning procedures. HEK293 cells transiently expression Cas9 via plasmid transfection was used as a positive control. (**j-n**) Uncropped Western blots of those shown in Supplementary Fig. 4a. As for e-h, blots were cut after transfer to facilitate probing of multiple antibodies on the same membrane and reprobing with different histone/histone mark antibodies from different species was performed. (**o**) Uncropped Western blots of images in Supplementary Fig. 4e. Actin and NOTCH1 were reprobed on the same blot using different fluorescence-tagged antibodies. The image from the channel showing actin signal is shown to the right of the NOTCH1 image.

Supplementary Tables

Supplementary Table 1. Characterization summary of pediatric glioma, control, and gene-edited cells lines. Summary of phenotypic properties, H3F3A mutation status, and expression of various genes associated with H3.3 mutation for all cell lines in our study. Relative RNA and protein expression levels determined by qPCR and Western blots, respectively, are tabulated as mean ± sd with n=3. Cells were obtained from: (1) ScienCell, Cat #1840, (2) Dr. C. David Allis, (3) Dr. Michelle Monje, (4) Dr. Angel Carcaboso
**Denotes protein expression levels that are significantly different ($p<0.05$ for two-tailed student t-test) comparing HA to pediatric DIPGs or matched unedited parental lines to CRISPR gene-edited lines.*

	**Control lines**		**CRISPR gene-edited lines**					
	**HA (1)**	**SF188 (2)**	**HA-G34R**	**HA-K27M**	**SF-G34R**	**SF-K27M**	**XIII-WT**	**XVII-WT**
**Tumor type**	normal astrocytes (brain stem)	glioma	NA	NA	NA	NA	NA	NA
***H3F3A* gene status**	WT	WT	K27M	K27M	G34R	G34R	WT	WT
**Phenotype in culture**	adherent monolayer	adherent monolayer	tumorsphere	Semi-adherent	tumorsphere	tumorsphere	Semi-adherent	adherent
***H3F3A* (RNA)**	1.0 ± 0.0	0.7 ± 0.0	0.9 ± 0.1	1.5 ± 0.1	1.0 ± 0.1	2.5 ± 0.1	1.1 ± 0.2	1.0 ± 0.0
***H3F3B* (RNA)**	1.0 ± 0.1	1.9 ± 0.2	1.4 ± 0.1	1.3 ± 0.1	1.8 ± 0.1	1.9 ± 0.2	1.6 ± 0.2	1.4 ± 0.1
**H3.3 (protein)**	1.0 ± 0.1	2.3 ± 0.2*	1.9 ± 1.3	10.4 ± 7.3*	1.2 ± 0.9	3.9 ± 2.4*	2.2 ± 1.7	1.8 ± 1.8
***MYC* (RNA)**	1.0 ± 0.2	0.6 ± 0.1	1.4 ± 0.1	7.3 ± 0.2	2.3 ± 0.2	7.0 ± 0.5	4.5 ± 0.8	3.8 ± 0.5
**MYC (protein)**	1.0 ± 0.0	2.4 ± 1.5	5.2 ± 1.4*	5.0 ± 1.4*	8.1 ± 3.4	4.7 ± 0.7*	1.8 ± 1.2	1.4 ± 0.1
***MYCN* (RNA)**	1.0 ± 0.2	0.1 ± 0.0	7.5 ± 2.0	9.8 ± 0.8	12.3 ± 2.3	19.7 ± 1.1	10.8 ± 2.1	3.7 ± 0.8
**MYCN (protein)**	1.0 ± 0.0	1.9 ± 0.1*	5.9 ± 2.2*	2.8 ± 0.7*	9.0 ± 6.1	3.1 ± 1.8	1.5 ± 0.7*	1.2 ± 0.1*
***DCX* (RNA)**	1.0 ± 0.5	0.6 ± 0.1	7.2 ± 7.1	10.7 ± 9.0	8.8 ± 3.2	3.3 ± 1.9	11.5 ± 6.7	9.9 ± 6.3
***GRIA4* (RNA)**	1.0 ± 1.4	0.9 ± 1.4	65.8 ± 40.2	72.0 ± 49.7	63.1 ± 29.4	18.6 ± 11.6	130.2 ± 71.7	73.5 ± 43.7
***RAP1GAP* (RNA)**	1.0 ± 2.3	0.6 ± 0.4	10.8 ± 6.4	11.6 ± 8.2	12.2 ± 5.1	3.5 ± 2.2	17.6 ± 10.5	20.4 ± 13.3
***ATCAY* (RNA)**	1.0 ± 2.7	0.3 ± 0.1	10.3 ± 5.7	8.9 ± 6.1	20.0 ± 14.1	5.4 ± 4.2	25.5 ± 18.0	14.2 ± 11.8
***ASCL1* (RNA)**	1.0 ± 0.5	1.2 ± 0.1	146.0 ± 20.8	48.2 ± 16.7	153.3 ± 22.6	61.6 ± 13.7	292.0 ± 44.9	85.0 ± 15.5
***HES5* (RNA)**	1.0 ± 0.5	1.2 ± 0.1	9.6 ± 6.1	41.4 ± 10.0	12.4 ± 3.1	5.0 ± 4.5	20.0 ± 4.9	66.7 ± 16.4
***DNER* (RNA)**	1.0 ± 0.8	5.4 ± 1.6	23.6 ± 4.3	14.0 ± 3.4	30.1 ± 6.5	11.0 ± 1.8	36.6 ± 11.4	19.6 ± 4.7
***NOTCH1* (RNA)**	1.0 ± 6	1.2 ± 0.2	6.7 ± 1.2	3.6 ± 0.7	7.0 ± 1.3	2.9 ± 0.3	8.7 ± 2.1	5.9 ± 1.0

Supp. Table 1 continued	Pediatric DIPG lines							
	**DIPG-XIII (3)**	**DIPG-XVII (3)**	**DIPG-7 (4)**	**DIPG-8 (4)**	**DIPG-11 (4)**	**DIPG-14 (4)**	**DIPG-17 (4)**	**DIPG-19 (4)**
**Tumor type**	DIPG	DIPG	GBM	Fibrillary astrocytoma	Anaplastic astrocytoma	Anaplastic astrocytoma	Anaplastic astrocytoma	Anaplastic astrocytoma
***H3F3A* gene status**	K27M	K27M	K27M	K27M	K27M	K27M	K27M	K27M
**Phenotype in culture**	tumorsphere	tumorsphere	tumorsphere	dense fibrils, adherent	tumorsphere	tumorsphere	tumorsphere	tumorsphere
***H3F3A* (RNA)**	1.2 ± 0.0	0.6 ± 0.0	0.6 ± 0.0	1.3 ± 0.0	1.0 ± 0.0	0.4 ± 0.0	1.0 ± 0.1	1.0 ± 0.0
***H3F3B* (RNA)**	3.2 ± 0.2	2.2 ± 0.0	0.8 ± 0.0	1.3 ± 0.0	2.6 ± 0.3	0.6 ±. 0.0	2.0 ± 0.1	2.1 ± 0.0
**H3.3 (protein)**	4.4 ± 0.3*	11.4 ± 0.3*	5.5 ± 0.1*	6.2 ± 0.4*	4.1 ± 0.2*	2.4 ± 0.5	1.3 ± 0.2	2.4 ± 0.6
***MYC* (RNA)**	12.8 ± 1.0	5.4 ± 0.5	1.2 ± 0.1	12.0 ± 0.6	0.1 ± 0.0	0.8 ± 0.0	2.3 ± 0.1	0.2 ± 0.0
**MYC (protein)**	3.6 ± 1.6	1.9 ± 1.0	2.5 ± 1.4	4.8 ± 3.2	1.1 ± 0.7	0.8 ± 0.6	1.3 ± 0.8	1.0 ± 0.7
***MYCN* (RNA)**	46.2 ± 12.4	8.6 ± 1.1	1.9 ± 1.2	6.3 ± 2.1	13.6 ± 3.3	6.6 ± 1.7	12.6 ± 2.3	7.6 ± 2.0
**MYCN (protein)**	4.7 ± 1.3	2.8 ± 0.2*	2.3 ± 0.4*	3.9 ± 1.9	1.9 ± 0.6	1.8 ± 1.0	2.1 ± 0.2*	1.7 ± 0.2*
***DCX* (RNA)**	29.4 ± 5.3	33.9 ± 18.9	0.4 ± 0.2	182.3 ± 24.5	256.0 ± 36.6	26.4 ± 4.6	242.2 ± 17.3	70.0 ± 31.4
***GRIA4* (RNA)**	179.1 ± 95.4	250.7 ± 150.9	51.3 ± 11.2	657.1 ± 76.4	125.4 ± 8.2	81.6 ± 9.1	867.1 ± 95.0	147.0 ± 55.6
***RAP1GAP* (RNA)**	33.4 ± 7.0	68.6 ± 43.3	11.8 ± 2.3	171.3 ± 36.2	152.2 ± 20.8	77.7 ± 19.4	74.5 ± 13.2	47.2 ± 17.9
***ATCAY* (RNA)**	52.2 ± 36.5	54.6 ± 7.0	6.1 ± 1.3	156.5 ± 39.8	38.9 ± 6.1	41.1 ± 3.4	103.3 ± 19.7	22.3 ± 8.5
***ASCL1* (RNA)**	558.3 ± 68.6	210.8 ± 28.3	13.1 ± 1.3	229.1 ± 48.3	268.7 ± 17.7	206.5 ± 11.8	167.7 ± 17.4	93.7 ± 8.3
***HES5* (RNA)**	47.5 ± 10.7	229.9 ± 29.0	0.3 ± 0.0	81.0 ± 18.8	498.0 ± 51.8	81.0 ± 6.8	64.4 ± 5.7	2.1 ± 0.4
***DNER* (RNA)**	85.6 ± 12.5	51.8 ± 11.2	1.1 ± 0.6	133.4 ± 37.3	284.0 ± 18.2	80.0 ± 8.4	183.5 ± 19.1	54.9 ± 3.9
***NOTCH1* (RNA)**	18.6 ± 4.4	13.2 ± 2.1	6.4 ± 0.6	8.9 ± 2.0	16.3 ± 1.6	11.8 ± 0.7	15.0 ± 1.3	11.2 ± 0.7

Supplementary Table 2. Summary of MACS2 and DiffBind results for H3K27me3 and H3.3 ChIP-Seq samples.

H3K27me3 ChIP-Seq Analysis:

Sample	Peaks
XIII rep1	66665
XIII rep2	67886
XIII-WT rep1	156729
XIII-WT rep 2	159585
XVII rep1	164917
XVII rep2	90759
XVII-WT rep1	171091
XVII-WT rep2	173572

DiffBind Analysis:

Sample	Unique peaks
XIII	21010
XIII-WT	87758
XVII	5190
XVII-WT	27049

H3.3 ChIP-Seq Analysis:

Sample	Peaks
XIII rep1	153096
XIII rep2	146972
XIII-WT rep1	149526
XIII-WT rep 2	153327
XVII rep1	158220
XVII rep2	159952
XVII-WT rep1	173147
XVII-WT rep2	166563

DiffBind Analysis:

Sample	Unique Peaks
XIII + XVII	1581
XIII-WT + XVII-WT	7488

Supplementary Table 3. Summary of MEME motif analysis of top 1000 regions with differential H3.3 or H3K27me3 signal for each cell line.

Enriched motifs in the top 1000 most differentially bound H3.3 peaks between WT and mutant cells for each line.

H3K27me3 Sample	Total peaks
XIII-WT only	87758
XIII (mutant) only	21010
XVII-WT only	27049
XVII (mutant) only	5190

Enriched motifs in the top 1000 most differentially bound H3K27me3 peaks between WT and mutant cells for each line.

HA	HA-G34R	HA-K27M	SF188	SF-G34R
2.32	2.48	1.27	2.17	1.29
1.55	1.40	0.23*	1.48	0.95*
1.55	1.40	0.23*	1.48	0.95*
1.55	1.40	0.23*	1.48	0.95*

Supplementary Table 4. Overlap of regions with changes in H3K27me3 and H3.3 levels in Line XIII and Line XVII.

Overlap of H3K27me3 and WT-only H3.3 peaks:

H3K27me3 Sample	Total peaks	Overlap
XIII-WT only	87758	80
XIII (mutant) only	21010	171
XVII-WT only	27049	79
XVII (mutant) only	5190	65

Overlap of H3K27me3 and mutant-only H3.3 peaks:

H3K27me3 Sample	Total peaks	Overlap
XIII-WT only	87758	131
XIII (mutant) only	21010	1
XVII-WT only	27049	27
XVII (mutant) only	5190	0

Supplementary Table 5. Overlap of gene expression changes and H3K27me3 or H3.3 peak changes between WT and K27 mutant cells.

Line XIII	WT up genes	WT down genes
**WT H3K27me3 only peaks**	521	688

**Mut H3K27me3 only peaks**	182	72
**WT H3.3 only peaks***	151	67
**Mut H3.3 only peaks***	19	12

**Line XVII**	**WT up genes**	**WT down genes**
**WT H3K27me3 only peaks**	177	304
**Mut H3K27me3 only peaks**	81	45
**WT H3.3 only peaks***	101	71
**Mut H3.3 only peaks***	10	5

* H3.3 WT and Mut only peaks from combined Line XIII and Line XVII analysis

Supplementary Table 6. Drug IC50s of H3.3 reciprocally-edited cell line panel. See also Figure 5 and Figure S6. IC50 (μM) values are tabulated for each drug treatment with n=3. DAPT was additionally combined with 2GY radiation treatment, leading to pronounced sensitivity of H3.3-mutant cells. **Denotes values for which the corresponding logIC50s that are significantly different (p<0.05 for two-tailed student t-test) than matched unedited parental lines. Pano = Panobinostat*

	**HA**	**HA-G34R**	**HA-K27M**	**SF188**	**SF-G34R**	**SF-K27M**	**XIII**	**XIII-WT**	**XVII**	**XVII-WT**
**DAPT**	2.32	2.48	1.27	2.17	1.29	0.74	1.30	2.37*	0.64	1.85*
**DAPT+ radiation**	1.55	1.40	0.23*	1.48	0.95*	1.04	0.47	2.08*	0.38	1.23*
**Pano**	0.13	0.19	0.11	0.10	0.14	0.09	0.09	0.08	0.08	0.08

Supplementary References

1. Harutyunyan, A. S. *et al.* H3K27M induces defective chromatin spread of PRC2-mediated repressive H3K27me2/me3 and is essential for glioma tumorigenesis. *Nat. Commun.* **10**, 1262 (2019).
