# Supplementary material for: Reciprocal H3.3 gene editing identifies K27M and G34R mechanisms in pediatric glioma including NOTCH signaling
Source: Commun Biol. 2020 Jul 9;3:363. doi: 10.1038/s42003-020-1076-0 (PMC7347881; doi:10.1038/s42003-020-1076-0)
Supplement: Supplementary file 2 — Description of Additional Supplementary Files [file 42003_2020_1076_MOESM2_ESM.pdf]

Descriptions of Additional Supplementary Files

Supplementary Data 1: Summary of MEME motif analysis of top 1000 regions with differential H3K27me3 or H3.3 signal for each cell line.

Supplementary Data 2: Lists of SE-linked genes that show differential expression in the Tag-seq data and overlap WT specific H3K27me3 peaks. Analysis divided by cell line (XIII or XVII) and direction of gene expression change in Tag-seq data.

Supplementary Data 3: Histology analysis and scoring of mouse brain sections of mice xenografted with our various H3.3 mutant and WT cell lines.

Supplementary Data 4: Raw and analyzed data for plots in main figures.
