# Supplementary material for: Reciprocal H3.3 gene editing identifies K27M and G34R mechanisms in pediatric glioma including NOTCH signaling
Source: Commun Biol. 2020 Jul 9;3:363. doi: 10.1038/s42003-020-1076-0 (PMC7347881; doi:10.1038/s42003-020-1076-0)
Supplement: Supplementary file 7 — Reporting Summary [file 42003_2020_1076_MOESM7_ESM.pdf]

nature research

Corresponding author(s): Paul Knoepfler

Last updated by author(s): May 27, 2020

Reporting Summary

Nature Research wishes to improve the reproducibility of the work that we publish. This form provides structure for consistency and transparency in reporting. For further information on Nature Research policies, see Authors & Referees and the Editorial Policy Checklist.

Statistics

For all statistical analyses, confirm that the following items are present in the figure legend, table legend, main text, or Methods section.

n/a	Confirmed	
☐	☒	The exact sample size (*n*) for each experimental group/condition, given as a discrete number and unit of measurement
☐	☒	A statement on whether measurements were taken from distinct samples or whether the same sample was measured repeatedly
☐	☒	The statistical test(s) used AND whether they are one- or two-sided<br>*Only common tests should be described solely by name; describe more complex techniques in the Methods section.*
☒	☐	A description of all covariates tested
☒	☐	A description of any assumptions or corrections, such as tests of normality and adjustment for multiple comparisons
☐	☒	A full description of the statistical parameters including central tendency (e.g. means) or other basic estimates (e.g. regression coefficient) AND variation (e.g. standard deviation) or associated estimates of uncertainty (e.g. confidence intervals)
☐	☒	For null hypothesis testing, the test statistic (e.g. *F*, *t*, *r*) with confidence intervals, effect sizes, degrees of freedom and *P* value noted<br>*Give P values as exact values whenever suitable.*
☒	☐	For Bayesian analysis, information on the choice of priors and Markov chain Monte Carlo settings
☒	☐	For hierarchical and complex designs, identification of the appropriate level for tests and full reporting of outcomes
☒	☐	Estimates of effect sizes (e.g. Cohen's *d*, Pearson's *r*), indicating how they were calculated

Our web collection on statistics for biologists contains articles on many of the points above.

Software and code

Policy information about availability of computer code

Data collection	ChIPseq: Illumina NovaSeq 6000, 150 base paired-end sequencing<br>3'-Tag-seq: Illumina HiSeq4000, 90 base single-end sequencing
Data analysis	ChIPseq: Burrows-Wheeler Aligner (BWA) was used for alignment. MACS2 was used to call peaks. Differential peak analysis was performed with the R package DiffBind.<br>3'-Tag-seq: alignment and differential expression analysis were performed using STAR-EdgeR and Tophat-Cufflinks. Gene ontology was analyzed with GOseq (Bioconductor version 3.8).<br>Drug dose-response and survival curve analysis: GraphPad Prism 5/6 softwares

For manuscripts utilizing custom algorithms or software that are central to the research but not yet described in published literature, software must be made available to editors/reviewers. We strongly encourage code deposition in a community repository (e.g. GitHub). See the Nature Research guidelines for submitting code & software for further information.

Data

Policy information about availability of data

All manuscripts must include a data availability statement. This statement should provide the following information, where applicable:

- Accession codes, unique identifiers, or web links for publicly available datasets
- A list of figures that have associated raw data
- A description of any restrictions on data availability

Raw and analyzed ChIP-seq and 3’-Tag-seq data are accessible upon publication via GEO (accession series# GSE129765). These data are plotted in Figures 2-4, Supplementary Figure 5, and Supplementary Tables 2-5.

Field-specific reporting

Please select the one below that is the best fit for your research. If you are not sure, read the appropriate sections before making your selection.

☒ Life sciences ☐ Behavioural & social sciences ☐ Ecological, evolutionary & environmental sciences

For a reference copy of the document with all sections, see nature.com/documents/nr-reporting-summary-flat.pdf

Life sciences study design

All studies must disclose on these points even when the disclosure is negative.

Sample size	Overall, in vitro cell assays were performed in biological triplicate at minimum. ChIP and Tag-seq were performed in biological duplicate. Xenograft studies were performed with N=6 mice for all interventions. This was determined using initial effect size data from a pilot experiment and calculated using power analysis with the software G*power.
Data exclusions	One ChIP-seq outlier was excluded due to low quality. These exlusion criteria were pre-established and all samples were screened with the same criteria.
Replication	All replications of experiments resulted in reproducible results.
Randomization	No covariates are analyzed in our study. Statistical comparisons were performed to compare H3.3WT and H3.3 mutant cell lines.
Blinding	Blinding was performed for the xenograft histology scoring performed by our collaborating cancer pathologist. Single blinding was conducted by providing numeric-coded samples without disclosing the mutation status to the pathologist.

Reporting for specific materials, systems and methods

We require information from authors about some types of materials, experimental systems and methods used in many studies. Here, indicate whether each material, system or method listed is relevant to your study. If you are not sure if a list item applies to your research, read the appropriate section before selecting a response.

Materials & experimental systems

n/a	Involved in the study	
☐	☒	Antibodies
☐	☒	Eukaryotic cell lines
☒	☐	Palaeontology
☐	☒	Animals and other organisms
☒	☐	Human research participants
☒	☐	Clinical data

Methods

n/a	Involved in the study	
☐	☒	ChIP-seq
☒	☐	Flow cytometry
☒	☐	MRI-based neuroimaging

Antibodies

Antibodies used	Primary antibodies – IgG (Santa Cruz, #sc-2027), mouse anti-c-Myc (Santa Cruz Biotechnology, #sc-40), mouse anti-n-Myc (Santa Cruz Biotechnology, #sc-53993), mouse anti-H3 (Upstate/EMD-Millipore, #05-499), rabbit anti-H3.3 (EMD-Millipore, #09-838), rabbit anti-H4 (Millipore Sigma, #04-858), rabbit anti-H3K27M (EMD-Millipore, #ABE419), mouse anti-Cas9 (Cell Signaling, #14697), rabbit anti-H3K27me3 (Cell Signaling, #C36B11), rabbit anti-H3.3S31p (AbCam #ab92628), rabbit anti-H3K27ac (Abcam, #ab4729), rabbit anti-K36me3 (Abcam, #ab9050), rabbit anti-NOTCH (Abcam, #ab52627), rabbit anti-ASCL1(MASH1) (Abcam, #ab74065), rabbit anti-RBPJ (Millipore-Sigma #ABE384), mouse anti-beta actin (Sigma, #A1978), anti-human nuclei (EMD-Millipore, #MAB1281B) and anti-H3K27M (EMD-Millipore, #ABE419) ; Secondary antibodies - IRDye 680RD goat anti-mouse (926-68070, LiCor) and IRDye800CW goat anti-rabbit (926-32211, LiCor).
Validation	All antibodies used have validation statements on manufacturer's website for the species (human) and application (IP, WB, or IHC) for which we used these antibodies. Multiple relevant citations are also on manufacturer's website for these antibodies.

Eukaryotic cell lines

Policy information about cell lines

Cell line source(s)	Human astrocyte-brain stem purchased from ScienCell; SF188 gifted by Dr. C. David Allis. The Rockefeller University; SU-DIPG-XIII and SU-DIPG-XVII gifted by Dr. Michelle Monje, Stanford University. DIPG-7, -8, -11, -14, -17, -19 were provided by Dr. Angel Carcaboso, Hospital Sant Joan de Deu. XIII-WT, XVII-WT, HA-K27M, HA-G34, SF-K27M and SF-G34R were generated in our lab by CRISPR-Cas9 gene editing for H3.3.

Authentication	HA-bs purchased from ScienCell are isolated and shipped at passage 1. They were not additionally validated. SF188, SU-DIPG-XIII, SU-DIPG-XVII were characterized by STR analysis prior to receiving in our lab. These along with XIII-WT, XVII-WT, HA-K27M, HA-G34, SF-K27M and SF-G34R were Sanger sequenced to determine H3.3 mutation status.
Mycoplasma contamination	All cell lines were submitted for mycoplasma testing and tested negative.
Commonly misidentified lines (See ICLAC register)	N/A

Animals and other organisms

Policy information about studies involving animals; ARRIVE guidelines recommended for reporting animal research

Laboratory animals	NOD scid gamma mic, 5-7 weeks old, equal ratio of male and female were used for xenografting cell lines into their brains.
Wild animals	N/A
Field-collected samples	N/A
Ethics oversight	UC Davis Institutional Animal Care and Use Committee (IACUC) reviewed and approved this study.

Note that full information on the approval of the study protocol must also be provided in the manuscript.

ChIP-seq

Data deposition

☒ Confirm that both raw and final processed data have been deposited in a public database such as GEO.

☒ Confirm that you have deposited or provided access to graph files (e.g. BED files) for the called peaks.

Data access links
May remain private before publication.

available upon publication

Files in database submission

13_H3_3_rep1_peaks.narrowPeak
13_H3_3_rep2_peaks.narrowPeak
13_WT_H3_3_rep1_peaks.narrowPeak
13_WT_H3_3_rep2_peaks.narrowPeak
17_H3_3_rep1_peaks.narrowPeak
17_H3_3_rep2_peaks.narrowPeak
17_WT_H3_3_rep1_peaks.narrowPeak
17_WT_H3_3_rep2_peaks.narrowPeak
13_H3K27me3_rep1_peaks.narrowPeak
13_H3K27me3_rep2_peaks.narrowPeak
13_WT_H3K27me3_rep1_peaks.narrowPeak
13_WT_H3K27me3_rep2_peaks.narrowPeak
17_H3K27me3_rep1_peaks.narrowPeak
17_H3K27me3_rep2_peaks.narrowPeak
17_WT_H3K27me3_rep1_peaks.narrowPeak
17_WT_H3_3_rep1_peaks.narrowPeak
13_H3_3_rep1_S468_L004_R1_001.fastq.gz
13_H3_3_rep2_S469_L004_R1_001.fastq.gz
13_WT_H3_3_rep1_S470_L004_R1_001.fastq.gz
13_WT_H3_3_rep2_S471_L004_R1_001.fastq.gz
17_H3_3_rep1_S472_L004_R1_001.fastq.gz
17_H3_3_rep2_S473_L004_R1_001.fastq.gz
17_WT_H3_3_rep1_S474_L004_R1_001.fastq.gz
17_WT_H3_3_rep2_S475_L004_R1_001.fastq.gz
13_H3_3_rep1_S468_L004_R2_001.fastq.gz
13_H3_3_rep2_S469_L004_R2_001.fastq.gz
13_WT_H3_3_rep1_S470_L004_R2_001.fastq.gz
13_WT_H3_3_rep2_S471_L004_R2_001.fastq.gz
17_H3_3_rep1_S472_L004_R2_001.fastq.gz
17_H3_3_rep2_S473_L004_R2_001.fastq.gz
17_WT_H3_3_rep1_S474_L004_R2_001.fastq.gz
17_WT_H3_3_rep2_S475_L004_R1_001.fastq.gz
13_H3K27me3_rep1_S476_L004_R1_001.fastq.gz
13_H3K27me3_rep2_S477_L004_R1_001.fastq.gz
13_WT_H3K27me3_rep1_S478_L004_R1_001.fastq.gz
13_WT_H3K27me3_rep2_S479_L004_R1_001.fastq.gz
17_H3K27me3_rep1_S480_L004_R1_001.fastq.gz
17_H3K27me3_rep2_S481_L004_R1_001.fastq.gz
17_WT_H3K27me3_rep1_S482_L004_R1_001.fastq.gz

17_WT_H3K27me3_rep2_S483_L004_R1_001.fastq.gz
13_H3K27me3_rep1_S476_L004_R2_001.fastq.gz
13_H3K27me3_rep2_S477_L004_R2_001.fastq.gz
13_WT_H3K27me3_rep1_S478_L004_R2_001.fastq.gz
13_WT_H3K27me3_rep2_S479_L004_R2_001.fastq.gz
17_H3K27me3_rep1_S480_L004_R2_001.fastq.gz
17_H3K27me3_rep2_S481_L004_R2_001.fastq.gz
17_WT_H3K27me3_rep1_S482_L004_R2_001.fastq.gz
17_WT_H3K27me3_rep2_S483_L004_R2_001.fastq.gz
13_Input_S464_L004_R1_001.fastq.gz
13_Input_S464_L004_R2_001.fastq.gz
13_WT_Input_S465_L004_R1_001.fastq.gz
13_WT_Input_S465_L004_R2_001.fastq.gz
17_Input_S466_L004_R1_001.fastq.gz
17_Input_S466_L004_R2_001.fastq.gz
17_WT_Input_S467_L004_R1_001.fastq.gz
17_WT_Input_S467_L004_R2_001.fastq.gz

Genome browser session
(e.g. UCSC)

https://genome.ucsc.edu/s/Knoepfler%20Lab/H3K27me3_H3.3

Methodology

Replicates

ChIP-seq was performed in duplicate for each sample

Sequencing depth

Approximately 150 million reads were sequenced for each ChIP sample, of these between 75-95 percent aligned to the hg19 genome. Libraries were sequenced on the NovaSeq machine with 150 base paired-end sequencing.

Antibodies

rabbit anti-H3.3 (EMD-Millipore, #09-838), rabbit anti-H3K27me3 (Cell Signaling, #C36B11)

Peak calling parameters

MACS2 callpeaks command was used to call peaks with the default settings. An input sample was sequenced and used as the control file in peak calling for each ChIP

Data quality

Peaks above 5 fold enrichment with FDR .05 cut-off:
13 H3K27me3 rep1 22202
13 H3K27me3 rep2 11941
17 H3K27me3 rep1 14038
17 H3K2me3 rep2 3960
17 WT H3K27me3 rep1 23846
17 WT H3K27me3 rep2 26092
13 WT H3K27me3 rep1 27017
13 WT H3K27me3 rep2 29479
17 WT H3.3 rep2 14492
13 WT H3.3 rep1 17884
13 WT H3.3 rep2 19532
17 H3.3 rep1 21656
17 H3.3 rep2 21095
17 WT H3.3 rep1 17058
13 H3.3 rep1 20036
13 H3.3 rep2 18566

Software

Libraries were sequenced on the NovaSeq machine. Reads were called with Illumina bclfastq.
